# Factors Associated with HIV Infection in Married or Cohabitating Couples in Kenya: Results from a Nationally Representative Study

**DOI:** 10.1371/journal.pone.0017842

**Published:** 2011-03-15

**Authors:** Reinhard Kaiser, Rebecca Bunnell, Allen Hightower, Andrea A. Kim, Peter Cherutich, Mary Mwangi, Tom Oluoch, Sufia Dadabhai, Patrick Mureithi, Nelly Mugo, Jonathan Mermin, for the KAIS Study Group

**Affiliations:** 1 Center for Global Health, Division of Global HIV/AIDS, Centers for Disease Control and Prevention, Nairobi, Kenya; 2 Center for Global Health, Division of Global HIV/AIDS, Centers for Disease Control and Prevention, Atlanta, Georgia, United States of America; 3 National AIDS/STI Control Programme (NASCOP), Nairobi, Kenya; 4 University of California San Francisco, Nairobi, Kenya; 5 National AIDS Control Council (NACC), Nairobi, Kenya; 6 Kenyatta National Hospital, Nairobi, Kenya; 7 University of Nairobi, Nairobi, Kenya; 8 National Center for Chronic Disease Prevention and Health Promotion, Division of Adult and Community Health, Centers for Disease Control and Prevention, Atlanta, Georgia, United States of America; 9 Center for Global Health, Division of Parasitic Diseases and Malaria, Centers for Disease Control and Prevention, Atlanta, Georgia, United States of America; 10 National Center for HIV/AIDS, Viral Hepatitis, STD, and TB Prevention, Division of HIV/AIDS Prevention, Centers for Disease Control and Prevention, Atlanta, Georgia, United States of America; New England Biolabs, Inc., United States of America

## Abstract

**Background:**

In order to inform prevention programming, we analyzed HIV discordance and concordance within couples in the Kenya AIDS Indicator Survey (KAIS) 2007.

**Methods:**

KAIS was a nationally representative population-based sero-survey that examined demographic and behavioral indicators and serologic testing for HIV, HSV-2, syphilis, and CD4 cell counts in 15,853 consenting adults aged 15–64 years. We analyzed interview and blood testing data at the sexual partnership level from married or cohabitating couples. Multivariable regression models were used to identify factors independently associated with HIV discordant and concordant status.

**Results:**

Of 3256 couples identified in the survey, 2748 (84.4%) had interview and blood testing data. Overall, 3.8% of couples were concordantly infected with HIV, and in 5.8% one partner was infected, translating to 338,000 discordant couples in Kenya. In 83.6% of HIV-infected Kenyans living in married or cohabitating couples neither partner knew their HIV status. Factors independently associated with HIV-discordance included young age in women (AOR 1.5, 95% CI: 1.2–1.8; p<0.0001), increasing number of lifetime sexual partners in women (AOR 1.5, 95% CI: 1.3–1.8; p<0.0001), HSV-2 infection in either or both partners (AOR 4.1, 95% CI: 2.3–7.2; p<0.0001), and lack of male circumcision (AOR 1.6, 95% CI: 1.0–2.5; p = 0.032). Independent factors for HIV-concordance included HSV-2 infection in both partners (AOR 6.5, 95% CI: 2.3–18.7; p = 0.001) and lack of male circumcision (AOR 1.8, 95% CI: 1.0–3.3; p = 0.043).

**Conclusions:**

Couple prevention interventions should begin early in relationships and include mutual knowledge of HIV status, reduction of outside sexual partners, and promotion of male circumcision among HIV-uninfected men. Mechanisms for effective prevention or suppression of HSV-2 infection are also needed.

## Introduction

Sub-Saharan Africa has the highest prevalence and incidence of HIV infection worldwide, mostly attributable to heterosexual transmission [1]. In Africa, there is increasing evidence that a large proportion of new HIV infections occur in cohabitating couples [2], [3], many of whom are unaware of both partners' sero-status [4], [5]. In East Africa, 40–50% of married or cohabitating HIV-infected persons are in an HIV-discordant partnership [6]. In Kenya, Uganda and Malawi, over 80% of all unprotected sex acts by HIV-infected persons occur with spouses or cohabitating partners [7], [8]. Consequently, a high proportion of incident HIV infections occur within married or cohabitating heterosexual couples, e.g., in Uganda 65% (2004–5) [9] and in Zambia (2001–2) and Rwanda, an estimated 52–93% (2005) [3].

HIV transmission in couples has been associated with high HIV viral load [10], lack of male circumcision [11], extramarital sex [5], low literacy [12], ignorance of self or partner's HIV status [8], limited understanding that HIV discordance can exist within couples [13], and other sexually transmitted infections [14], [15]. Transmission among couples has been reduced by 80% with effective interventions, including couple-specific counseling and testing and condom provision [4]. In addition, modeling suggests that HIV transmission in heterosexual partnerships is reduced by antiretroviral treatment (ART) [16], and there are a number of observational studies that have shown this empirically [17], [18]. However, little information is available on the risk factors for HIV transmission among couples on a national level.

We analyzed couples data from a nationally-representative, population-based survey conducted in Kenya in 2007 that included self-reported data on demographics, sexual behaviors, male circumcision, previous HIV testing, HIV status, ART use, and laboratory testing results for HIV, herpes simplex virus type 2 (HSV-2), syphilis, and CD4 counts for HIV-infected respondents. To help inform prevention interventions, we assessed independent factors associated with HIV discordance compared to HIV-uninfected couples and associated with HIV-infected concordance compared to HIV discordant couples.

## Methods

### Ethics statement

Participants provided separate informed oral consent for interviews, blood draws and blood storage. Survey protocols were approved by the Ethics Review Committee of the Kenya Medical Research Institute (KEMRI) and the Institutional Review Board of the U.S. Centers for Disease Control and Prevention (CDC).

### Study design

The Kenya AIDS Indicator Survey (KAIS) was a nationally representative, population-based household survey involving persons aged 15–64 years. The design for KAIS was a stratified, two-stage cluster sample that was for comparable with the design of the 2003 Kenya Demographic and Health Survey (DHS). The first stage involved selecting clusters from the same sampling frame that was used for the 2003 DHS, based on the 1999 national census, and the second stage involved the selection of households per cluster with equal probability of selection in the rural-urban strata within each district. A total of 29 field teams, each consisting of six data collectors (four interviewers and two laboratory technicians), one supervisor and one driver, conducted fieldwork from August to December 2007. Teams administered questionnaires in local languages where necessary, in addition to questionnaires in Kiswahili and English, to accommodate respondents that were not conversant in vernacular languages. All questionnaires were back translated into English. Survey personnel participated in intensive two week training in KAIS procedures. All questionnaire responses were double-entered using Census and Survey Processing System (CSPro) version 3.3 (U.S. Census Bureau. Washington DC, USA). A series of internal consistency and range checks helped to identify any illogical responses and to verify that responses adhered to skip patterns in the questionnaire. Demographic and HIV/AIDS-specific indicators included HIV testing history, self-reported HIV status, and partner-specific behaviors and disclosure. Blood specimens were obtained in households for testing at the national reference laboratory in Nairobi for HIV, HSV-2 and syphilis. Individual test results were linked to individual and household questionnaires through unique identification numbers.

### Laboratory methods

Vironostika HIV Uni-Form II antigen/antibody (BioMérieux Bv, Boseind, Netherlands) and Murex HIV antigen/antibody (Abbott/Murex-Biotech Ltd, Kent, UK) tests were used for HIV screening and confirmation, respectively, in a serial testing algorithm. Discordant samples were retested with the two assays and polymerase Chain Reaction (PCR) (Roche HIV DNA v 1.5) tests were conducted on all samples with two sets of discordant results. For quality control, all positive specimens and a random sample of 5% of negative specimens was retested in a different laboratory using the same testing algorithm. The Kalon HSV Type 2- specific IgG EIA was used for HSV-2 testing. This was a recombinant type 2 antigen (gG2) modified to eliminate reactivity arising from HSV type 1 infection and at the same time retaining the natural antigenic characteristics of HSV-2. For syphilis testing, the *Treponema pallidum* particle agglutination (TPPA) assay was used as a screening test and rapid plasma reagin (RPR) for confirmation at a dilution of 1∶1.

### Measures

Couples were defined as being in a married or cohabitating partnership between the male head of household and his wife. In case of a polygamous partnership the husband and only one partner were included (n = 298 couples). A couple was considered HIV-discordant if one partner was HIV-infected and the other partner HIV-uninfected in HIV testing done during the study. A couple was considered to be concordantly uninfected if neither partner was HIV-infected and considered concordantly HIV-infected if both partners were HIV-infected. Couples were considered HIV-affected when they were either HIV-discordant or -concordant. We developed 2 different models to assess factors associated with couple status – one for HIV discordance as the outcome and a second one with HIV concordance as the binary outcome. In the first model, concordant HIV-infected couples were excluded from the denominator and in the second model concordant uninfected couples were excluded from the denominator. Predictor variables included socio-demographic characteristics, age difference between partners, length of relationship in years, male circumcision, syphilis, HSV-2, CD4 cell count, perception of HIV risk, outside partners, consistent condom use with outside partner in the last 12 months, number of lifetime sexual partners, and desire to have a child within next two years among women. All predictor variables were self-reported except for CD4 cell count of infected partners, HSV-2 and syphilis status. Male circumcision was based on self-report as physical examinations were not conducted. Correct knowledge of HIV status was defined as self-reported HIV status validated by laboratory testing during the study. Low perception of HIV risk was defined as small or no self-reported risk for HIV.

### Statistical analysis

We performed all analyses in SAS version 9.1 (SAS Institute Inc., Cary, North Carolina, USA). Using multivariable logistic regression (SAS Survey Logistic), we calculated adjusted odds ratios (AOR) and 95% confidence intervals (CI) to identify variables associated independently with each primary outcome. Variables that were statistically significant at a 0.05 p-value level in bivariate analyses were selected for the final multivariable model by use of backwards elimination if they did not remain significant. Two way interactions between variables were considered. Confidence intervals for percentages were computed. Logistic regression was used to assess whether the probability of having a relationship of 20 years or more was related to knowledge of status and disclosure. Analyses accounted for the stratified cluster design of the survey. Each response was weighted to account for its sampling probability and to adjust for non-response rates. National estimates of numbers of concordant positive and discordant positive couples were derived by multiplying the estimated national population aged 15–64 years (19,319,000) by the estimated proportion of this group that was married/cohabitating based on survey results (0.604), then dividing by 2 to get the number of couples and finally multiplying that result by the estimated proportion of couples that were discordant (0.058) or concordant positive (0.038). Correct knowledge and disclosure of HIV status were not included in the models because of uncertainty of the cause-effect relationship in a cross-sectional study and because of our inability to construct a variable by HIV status to measure the prevention effect in the infected discordant partner. We also decided not to include condom use between married or cohabitating partners in the models because we were not able to adjust for correct knowledge and disclosure of HIV infection status. Widow-hood was not included in models because this information was not available for men and numbers were too small for women.

## Results

### Couple characteristics

**Figure 1:**
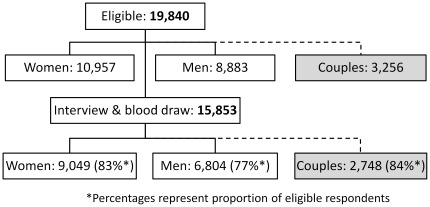
Eligibility, response rates and available married or cohabitating couple data, Kenya AIDS Indicator Survey 2007.

A total of 9,049 women and 6,804 men aged 15–64 years living in 9,691 households from 402 clusters participated in both interviews and blood sample collection. This included 2,748 married or cohabitating couples. The response rate for couples was 84.4% (Figure 1). Overall, 9.6% (95% CI: 8.3–10.9) of couples were affected by HIV; 3.8% (95% CI: 3.0–4.5) were concordantly infected and 5.8% discordantly infected (95% CI: 4.7–6.8). In 48.5% (95% CI: 40.3–56.5) of discordant couples the woman was the HIV-infected partner. Extrapolating to population estimates, this corresponds to an estimated 219,000 concordant infected couples and 338,000 discordant couples in Kenya. Of all couples, 30.4% (95% CI: 28.3–32.5) were concordantly positive for HSV-2 and 20.7% (95% CI: 18.9–22.5) were HSV-2 discordant.

Among all married or cohabitating HIV-infected Kenyans, 42.8% (95% CI: 36.5–49.0) had a previous HIV test and 16.4% (95% CI: 11.5–20.8) knew their HIV status correctly. Among the 83.6% HIV-infected Kenyans living in a couple who did not know their status correctly, 24.8% (95% CI: 20.1–29.6) self-reported that they were HIV-uninfected based on their most recent test results, and 57.2% (95% CI: 51.0–63.5) never had an HIV test or received their test result. Only 14.9% (95% CI: 10.4–19.4%) of HIV-infected Kenyans living in a couple correctly knew their status and also reported that they disclosed to their partner; however, this meant that most disclosed when they knew their status. Among discordant couples, in 5.5% (95% CI: 1.7–9.3) both partners correctly knew their status and disclosed their status to the partner. In 31.8% of discordant couples the infected partner reported being HIV-uninfected based on a prior test. In infected concordant couples, 16.2% (95% CI: 10.0–22.4) correctly knew their status and disclosed their status to the partner. In 34.7% (95% CI: 26.5–42.9) of concordant couples at least one partner had a prior HIV-negative test and was unaware of his or her current HIV infection. Partnerships of 20 years or more duration were reported by 38.3% (95% CI: 29.3–47.3) of discordant and 28.4% (95% CI: 18.6–38.2) of concordant couples. Among those, rates of correct knowledge of status and disclosure were not significantly different than among couples with relationships of less than 20 years. In 3.2% uninfected (95% CI: 2.4–5.0), 6.6% discordant (95% CI: 2.5–10.6) and 16.3% concordant (95% CI: 9.2–23.5) infected couples one or both partners reported consistent condom use in the last 12 months.

Table 1 shows characteristics of married or cohabitating couples for predictor variables included in bivariate analyses. Proportions of HIV-affected couples were highest in the 15–24 years age categories for both female (4.1%, 95% CI: 1.9%–6.4%) and male (4.3%, 95% CI: 1.8%–6.9%) HIV-infected partners. Furthermore, the proportions of HIV-affected couples were highest in Nyanza (21.8%, 95% CI: 17.3%–26.4%) and lowest in North Eastern (2.8%, 95% CI: 0%–5.8%) (Table 1), the provinces with the highest and lowest HIV prevalence rates (14.9% and 0.8%, respectively [19]).

**Table 1:** Characteristics of married or cohabitating couples, Kenya AIDS Indicator Survey 2007.

		All couples	HIV discordance, woman infected, man uninfected	HIV discordance, woman uninfected, man infected	HIV-infected concordance	HIV uninfected concordance
Characteristics	Categories	Unweighted Frequency	Weighted Percentage	Weighted Percentage	Weighted Percentage	Weighted Percentage
	**Total**	2748	2.8	3.0	3.8	90.4
**Age, men (n = 2748)**	Men, 15–24	104	1.0	1.6	1.2	96.3
	Men, 25–29	289	3.4	4.3	6.2	86.1
	Men, 30–39	850	4.1	2.9	4.8	88.2
	Men, 40–49	716	2.7	3.8	3.6	89.9
	Men, 50–59	555	1.3	2.1	2.7	93.8
	Men, 60–64	234	1.9	1.7	1.0	95.4
**Age, women (n = 2748)**	Women, 15–24	484	3.4	5.2	4.8	86.7
	Women, 25–29	500	4.1	2.1	5.5	88.3
	Women, 30–39	877	3.6	3.2	4.8	88.4
	Women, 40–49	576	0.8	2.9	1.0	95.3
	Women, 50–59	280	1.8	0.3	1.4	96.6
	Women, 60–64	31	0	2.3	5.2	92.5
**Residence (n = 2748)**	Rural	2233	2.7	2.8	3.7	90.8
	Urban	515	3.1	4.1	4.1	88.6
**Province (n = 2748)**	Nairobi	208	4.4	3.4	6.1	86.1
	Central	384	1.7	1.6	2.0	94.6
	Coast	289	2.7	4.1	2.1	91.0
	Eastern	425	2.9	1.5	1.9	93.7
	North Eastern	152	1.0	1.3	0.5	97.2
	Nyanza	426	4.6	6.6	10.6	78.2
	Rift Valley	443	2.9	2.6	2.6	91.9
	Western	421	1.2	2.2	2.3	94.2
**Education, men (n = 2748)**	Men, no education	318	1.3	0.9	1.4	96.4
	Men, primary incomplete	666	1.9	3.5	3.8	90.8
	Men, primary complete	762	3.2	2.3	5.5	89.0
	Men, secondary +	1002	3.5	3.7	2.9	89.9
**Education, women (n = 2748)**	Women, no education	494	0.7	1.6	1.0	96.6
	Women, primary incomplete	855	3.3	3.2	5.0	88.5
	Women, primary complete	740	3.0	3.3	4.8	89.0
	Women, secondary +	659	3.1	3.0	2.5	91.3
**Marital Status (n = 2743)**	Monogamous	2445	2.8	3.0	3.7	90.4
	Polygamous	298	2.5	2.8	4.3	90.4
**Age difference between partners (n = 2748)**	<5 years	958	2.1	2.5	4.1	91.3
	men 5–9 yrs older	1073	3.0	2.9	3.4	90.7
	men 10+ yrs older	717	3.4	3.9	4.0	88.8
**Length of relationship in years (n = 2547)**	0–9	784	3.6	3.2	5.8	87.4
	10–19	647	3.3	2.6	3.9	90.2
	20–29	769	1.6	3.9	2.9	91.6
	30+	347	2.2	1.9	1.7	94.2
**Male Circumcision (n = 2738)**	Yes	2400	2.7	2.2	2.2	92.8
	No	338	3.2	8.4	13.6	74.8
**Syphilis (n = 2695)**	Both man and woman are syphilis-infected	4	0.0	0.0	0.0	100.0
	The man is syphilis-infected, but not the woman	51	3.4	7.3	12.6	76.7
	The woman is syphilis-infected, but not the man	29	9.3	2.7	2.1	85.9
	Neither man nor woman are syphilis-infected	2611	2.7	2.9	3.6	90.7
**HSV-2 (n = 2704)**	Both man and woman are HSV-2-infected	775	4.4	5.1	10.1	80.5
	The man is HSV-2-infected, but not the woman	207	2.1	6.0	0.6	91.3
	The woman is HSV-2-infected, but not the man	368	6.3	1.9	3.5	88.3
	Neither man nor woman are HSV-2-infected	1354	1.0	1.6	0.4	97.1
**Perception of HIV risk (n = 2255)**	Both man nor woman have a perception of low HIV risk	1515	1.9	2.8	1.5	93.7
	The man has a perception of low HIV risk, but not the woman	420	4.6	2.1	4.6	88.7
	The woman has a perception of low HIV risk, but not the man	200	3.5	4.4	2.9	89.2
	Neither man or woman have a perception of low HIV risk	120	2.5	3.3	7.9	86.3
**Having outside partners (n = 2746)** *	Neither has outside partners	2562	2.6	3.0	3.8	90.7
	The man, but not the woman, has outside partners	164	6.4	2.7	2.4	88.4
	The woman, but not the man, has outside partners	20	3.0	8.3	14.0	74.7
**Consistent condom use with outside partners in the last 12 months (n = 2748)** *	Neither the man nor the woman reported using condoms with outside partners in the last 12 months	2690	2.7	3.0	3.8	90.6
	Either the man or the woman reported using condoms with outside partners in the last 12 months	58	9.0	4.1	1.8	85.1
**Number of lifetime sexual partners, men (n = 2512)**	Men who report 1 lifetime partner	258	1.0	1.5	1.8	95.7
	Men who report 2–3 lifetime partner	736	2.9	1.8	2.8	92.4
	Men who report 4+ lifetime partner	1518	2.8	3.2	3.9	90.0
**Number of lifetime sexual partners, women (n = 2705)**	Women who report 1 lifetime partner	1176	0.8	1.5	1.4	96.3
	Women who report 2–3 lifetime partner	1258	3.6	4.2	4.8	87.4
	Women who report 4+ lifetime partner	271	6.2	3.1	7.7	83.0
**Women who reported desire to have child within next 2 years (n = 2465)**	Yes	250	5.9	3.9	5.5	84.7
	No	2215	2.6	3.0	3.7	90.7

*Only in 3 couples both partners reported having outside partners; category was set missing.

### Factors associated with discordance

Table 2 shows results of bivariate and multivariable analyses for variables associated with HIV discordance compared to HIV uninfected concordance. In final multivariable models, predictors for HIV-discordance included younger age in women (AOR 1.5, 95% CI: 1.2–1.8 per 10 years; p<0.0001), increasing number of lifetime sexual partners in women (AOR 1.5, 95% CI: 1.3–1.8; p<0.0001), HSV-2 infection in either (man infected: AOR 3.1, 95% CI: 1.6–6.3; p = 0.001; woman infected: AOR 3.5, 95% CI: 1.9–6.4; p<0.0001) or both partners (AOR 4.1, 95% CI: 2.3–7.2; p<0.0001), and lack of male circumcision (AOR 1.6, 95% CI: 1.0–2.5; p = 0.032) (Table 2).

**Table 2:** Unadjusted* and adjusted odds ratios for factors associated with HIV discordance vs. HIV uninfected concordance, Kenya AIDS Indicator Survey 2007.

		Bivariate		Multivariable	
Variables	Categories	OR (95% CI)	P value	AOR (95% CI)	P value
**Age, women**	Per 10 years of age younger	1.5 (1.2–1.8)	<.0001	1.5 (1.2–1.8)	<.0001
**Education, women**	No primary	Reference		n/a	
	Incomplete Primary	2.7 (1.3–5.7)	0.0083	n/a	n/a
	Complete Primary	2.5 (1.1–5.3)	0.0218	n/a	n/a
	Secondary or higher	2.5 (1.2–5.2)	0.0154	n/a	n/a
**Education, men**	No primary	Reference		n/a	
	Incomplete Primary	2.3 0.9–5.8	0.0746	n/a	n/a
	Complete Primary	2.3 0.9–5.8	0.0847	n/a	n/a
	Secondary or higher	2.9 (1.2–7.1)	0.0222	n/a	n/a
**Number of lifetime sexual partners, women**	Per additional partner	1.7 (1.4–2.0)	<.0001	1.5 (1.3–1.8)	<.0001
**Age Difference**	Male less than 5 years older	Reference		n/a	
	Male 5–9 years older	1.4 (0.9–2.0)	0.1598	n/a	n/a
	Male 10 or more years older	1.6 (1.1–2.4)	0.0246	n/a	n/a
**Having outside partners**	Neither	Reference		n/a	
	Man did, Woman did not	1.8 (1.0–3.2)	0.0500	n/a	n/a
	Man did not, Woman did	2.8 (0.7–11.8)	0.1515	n/a	n/a
**HSV-2**	Neither man nor woman are HSV-2-infected	Reference		Reference	
	The man is HSV-2-infected, but not the woman	3.3 (1.7–6.4)	0.001	3.1 (1.6–6.3)	0.001
	The woman is HSV-2-infected, but not the man	3.6 (2.0–6.3)	<.0001	3.5 (1.9–6.4)	<.0001
	Both man and woman are HSV-2-infected	4.2 (2.6–7.1)	<.0001	4.1 (2.3–7.2)	<.0001
**Male Circumcision**	Yes	Reference		Reference	
	No	2.8 (1.9–4.2)	<.0001	1.6 (1.0–2.5)	0.032
**Female partner wants a child within 2 years**	No	Reference		n/a	
	Yes	1.9 (1.1–3.5)	0.0247	n/a	n/a

*Table includes all variables that were significantly associated with HIV discordance vs. HIV uninfected concordance in the bivariate analysis.

### Factors associated with concordance

Table 3 shows results of bivariate and multivariable analyses for variables associated with HIV concordance compared to HIV discordance. In final multivariable models, independent factors for HIV-concordance included HSV-2 infection in both partners (AOR 6.5, 95% CI: 2.3–18.7; p = 0.001) and lack of male circumcision (AOR 1.8, 95% CI: 1.0–3.3; p = 0.043) (Table 3).

**Table 3:** Unadjusted* and adjusted odds ratios for factors associated with HIV-infected concordance vs. HIV discordance, Kenya AIDS Indicator Survey 2007.

		Bivariate		Multivariable	
Variables	Categories	OR (95% CI)	P value	AOR (95% CI)	P value
**Consistent condom use with outside partners in the last 12 months**	Either the man or the woman reported using condoms with outside partners	Reference		n/a	
	Neither the man nor the woman reported using condoms with outside partners	2.8 (1.2–6.3)	0.0151	n/a	n/a
**HSV-2**	Neither man nor woman are HSV-2-infected	Reference		Reference	
	The man is HSV-2-infected, but not the woman	0.5 (0.1–4.8)	0.549	0.6 (0.1–6.1)	0.657
	The woman is HSV-2-infected, but not the man	2.8 (0.9–8.8)	0.076	2.7(0.8–8.9)	0.114
	Both man and woman are HSV-2-infected	6.7 (2.5–18.0)	<.0001	6.5 (2.3–18.7)	0.001
**Male Circumcision**	Yes	Reference		Reference	
	No	2.6 (1.5–4.4)	<.0001	1.8 (1.0–3.3)	0.043

*Table includes all variables that were significantly associated with HIV-infected concordance vs. HIV discordance in the bivariate analysis.

No significant interactions were found in the models. Also, in both models there were too few people in partnerships with CD4 cell counts to use it as a proxy for length of infection or on ART to compute the effect of ART with any degree of confidence (e.g. there were 153 discordant couples with one partner on ART and available CD4 counts; of those, 24 had a CD4 count less than 250 cells/µl).

## Discussion

In a nationally representative sample of Kenyans aged 15–64, 1 in 10 married or cohabitating couples was affected by HIV. Regional variations were pronounced, with the highest proportion of couples in Nyanza province being HIV-affected and the lowest in North Eastern province. Nyanza province is located on the shore of Lake Victoria in the tropical part of Kenya with the highest rates for HIV and other infectious diseases in the country while North Eastern province is mostly arid and less populated. Younger age in women, an increasing number of lifetime partners in women, HSV-2 infection in one or both partners, and lack of circumcision in the male partner were associated with HIV infection in discordant couples compared with uninfected couples in multivariable analyses. Our findings suggest a scenario where young women may enter a marriage or cohabitation when they are already HIV-infected or HIV/HSV-2 –co-infected, especially if women have multiple sexual partners early after sexual debut. Young women may also be HIV-uninfected but start the relationship with a man who is already HIV-infected or HIV/HSV-2 –co-infected.

Despite considerably less power to show significant associations, HSV-2 infection in both partners and lack of circumcision in the male partner were associated with being in a concordantly infected couple compared to a discordant couple, indicating the need to address these risk factors in prevention programs for discordant couples. Our findings were consistent with the literature [4], [5], [8], [10]–[18] and therefore confirmed previously reported associations at a national population level in Kenya.

Married or cohabitating couples are a population at high risk for HIV transmission and acquisition in Kenya. Discordant couples represent a particular high risk group. Also, partners in the acute phase of a new infection pose a high risk for onward transmission within the couple or if they have unprotected sex outside of the couple. Without intervention, 8–12% of HIV-infected adults living in couples will transmit HIV to their partners annually [10]. If all the 338,000 uninfected partners became infected HIV prevalence in Kenya could increase by up to 2% (based on the KAIS 2007 prevalence of 7.1% [19]). Currently, prevention messages often ignore couples, focusing on casual partnerships despite the frequent lack of knowledge within HIV-affected couples of the risk of transmission within their partnership or through perinatal transmission. Lack of knowledge may be increased by a lack of risk awareness within a stable relationship that results in low condom use, as was confirmed in our study. The desire in female partners to have a child in the next 2 years did not remain significant as a risk factor for discordant couple status in the multivariable analysis; however, getting pregnant in a natural way requires unprotected sex, which raises the question of adequate risk counseling and family planning.

The importance of prevention interventions targeted to couples is increasingly recognized [3], [20]. Interventions need to encourage couples to be tested together early in the relationship and mutually know their HIV status by learning their test result at the same time or disclosing their status to each other. Partner testing for HIV-infected persons in care and treatment programs should be standard practice as recommended by the World Health Organization [21]. Our results indicated a considerable need for family planning counseling, which should be an integral part of all testing and counseling services. Understanding of HIV discordance, even among health providers, is limited, and common misconceptions may undermine risk reduction efforts [22]. Both lack of male circumcision and HSV-2 infection have been shown to be associated with increased HIV transmission [23]–[25]. Our findings confirm that male circumcision will benefit couples by increasing the male partner's likelihood of remaining HIV uninfected. HIV testing and counseling offer an opportunity to refer uncircumcised, HIV-uninfected men for voluntary medical male circumcision. Strong associations between HSV-2 infection and both HIV discordance and concordance reinforce the need to further advance our knowledge on the role of HSV-2 as a biological cofactor in HIV acquisition and transmission [25] and develop policies and program guidelines for HSV-2 as a risk factor for HIV infection in couples. HSV-2 suppression with twice daily acyclovir assessed in clinical trials did not prevent HIV acquisition [26], [27] or transmission [28]. An antiretroviral vaginal gel has recently shown promising results in reducing HSV-2 acquisition [29]. In addition, a HSV-2 vaccine, if it is approved, should be considered to reduce the risk of HSV-2 infection in HIV uninfected and discordant couples [30].

Similar proportions of couples as found in our study may be affected by HIV in other countries in Sub-Saharan Africa with a generalized HIV epidemic similar to Kenya's. The vast majority of these couples are unaware of their HIV infection and many perceive themselves to be at low risk with no need for testing. Opportunities for expanding couples testing and counseling exist in Kenya, including integration of partner testing into HIV care and treatment programs, enhanced partner testing in prevention of mother to child transmission and tuberculosis programs, and home-based HIV couples testing. Couples testing and counseling should be a central component of Kenya's national HIV prevention strategy.

Our analysis had some limitations. Cross-sectional surveys do not allow for determination of the sequence of cause and effect which complicates interpretation of associations. Because of the cross-sectional design, we were unable to include some factors associated with HIV acquisition in our models (e.g. correct knowledge of status). The way the household questionnaire was constructed only allowed including couples in which the head of household was the male partner and co-wives in polygamous partnerships could not be analyzed. Some data were not available from the study, e.g. whether respondents had tested for HIV as a couple. Finally, Kenya's population structure with over 40 ethnic tribes of considerable cultural differences may have resulted in some differences in self-reporting; however, the direction and magnitude of potential reporting bias is unknown.
